# Antibiotic prophylaxis for childbirth-related perineal trauma: A systematic review and meta-analysis

**DOI:** 10.1371/journal.pone.0323267

**Published:** 2025-05-09

**Authors:** Hannah Armstrong, Jane Whitehurst, R. Katie Morris, Victoria Hodgetts Morton, Rebecca Man, on behalf of the CHAPTER group

**Affiliations:** 1 Department of Applied Health Sciences, School of Health Sciences, College of Medicine and Health, University of Birmingham, Edgbaston, Birmingham, United Kingdom,; 2 Applied Research Collaboration, West Midlands, United Kingdom,; 3 Birmingham Women’s Hospital, Edgbaston, Birmingham, United Kingdom; Tehran University of Medical Sciences, IRAN, ISLAMIC REPUBLIC OF

## Abstract

**Introduction:**

Childbirth-related perineal trauma affects the vast majority of women who give birth vaginally and subsequent complications such as wound infection occur frequently. Antibiotic prophylaxis is not currently recommended following first or second degree tears, or episiotomies. We aimed to evaluate the effectiveness of prophylactic antibiotics for preventing complications from perineal trauma across all types of childbirth-related perineal trauma.

**Materials and methods:**

Databases were searched from inception to February 2024. Randomised controlled trials, non-randomised interventional studies and observational studies were eligible for inclusion where women experienced childbirth-related perineal trauma and received antibiotic prophylaxis or placebo, including any type of tear or episiotomy. The primary outcome was perineal wound infection. Results were combined into meta-analysis using a random effects model.

**Results:**

14 studies were eligible for inclusion (eight randomised controlled trials, six observational) involving 8,878 women. Ten studies were deemed to have a high overall risk of bias. Overall the GRADE certainty of findings were low. Twelve studies were included in the analysis for perineal wound infection, involving 8,438 women. There was a reduced rate of perineal wound infection when prophylactic antibiotics were received (relative risk 0.57, 95% confidence interval 0.48 to 0.67). The subgroup analysis by type of tear demonstrated association with reduced risk of infection when prophylactic antibiotics were received, in the non-obstetric anal sphincter injuries combined subgroup (relative risk 0.50, 95% confidence interval 0.41–0.63) and the episiotomy only subgroup (relative risk 0.57, 95% confidence interval 0.36–0.91).

**Conclusions:**

Prophylactic antibiotics are associated with a reduced risk of perineal wound infection. Despite these findings, there are not sufficient high-quality randomised controlled trials to adequately inform guideline change amongst women with first/second degree tears or episiotomies. We recommend that an adequately powered, robust, randomised controlled trial is needed amongst women with first/second degree tears or episiotomies after spontaneous birth.

## 1 Introduction

Perineal trauma is the most common complication after childbirth, affecting approximately 90% of nulliparous and 69% of multiparous women [1]. Childbirth-related perineal trauma (CRPT) can impact all aspects of a woman’s life, with some women suffering long-lasting physical and psychological complications [2,3]. Poor management of wound complications after CRPT can further reduce quality of life and lead to worsening long-term outcomes, such as postnatal depression, chronic pain, dyspareunia and incontinence [4]. Despite this high prevalence and the potential for long-term consequences, there is a lack of research on this topic.

The exact incidence of wound infections after CRPT is unknown and varies significantly across the literature, with estimates between 0.1–23.6% [5]. Whilst clear guidelines exist for the postnatal care of women who have sustained obstetric anal sphincter injury (OASI), there is a lack of evidence based guidelines for the postnatal care of non-OASI trauma [6]. Clinicians express a lack of confidence in managing CRPT in the postnatal period and women report feeling dissatisfied with their postnatal care [7,8]. The recent governmental Birth Trauma Inquiry Report released in 2024 highlights this, and the need to ensure that improving care in the postnatal period is prioritised by policy-makers, clinicians and researchers [9].

Studies investigating the use of prophylactic antibiotics for perineal trauma have found antibiotics to be effective for preventing infection after assisted vaginal birth and OASI [10,11], with current guidelines therefore recommending antibiotic prophylaxis following either of these events [6,12]. Antibiotic prophylaxis is, however, not currently recommended following non-OASI perineal trauma after spontaneous vaginal birth [13]─ there is consequently a need to consider the use of prophylactic antibiotics in these circumstances, considering both the effectiveness in reducing complications and any potential harms.

This review forms part of the CHAPTER (Childbirth Associated Perineal Trauma) programme grant, which overall aims to improve the care of women who sustain CRPT. Other outputs of the CHAPTER programme include the development of a wound assessment tool and care pathways for women who have experienced perineal trauma. Here, we aim to assess the effectiveness of prophylactic antibiotics for the prevention of complications in all types of CRPT.

## 2 Materials and methods

### 2.1 Study design and registration

This forms a systematic review and meta-analysis of studies evaluating prophylactic antibiotics compared to no prophylactic antibiotics for women with CRPT. The review was performed in-line with PRISMA guidance, and the review protocol was published prospectively under the overarching PROSPERO for the series of reviews conducted across the CHAPTER project (CRD42023458738) [14,15]. The conclusions drawn have been discussed and refined with our patient and public advisory CHAPTER co-applicant.

### 2.2 Inclusion and exclusion criteria

Randomised controlled trials (RCTs), non-randomised interventional studies and observational studies were eligible for inclusion, where women with perineal trauma following vaginal birth received prophylactic antibiotics, compared to no antibiotics or a placebo. We chose to include observational studies in order to collate all the available evidence, as from scoping searches we were aware the existing evidence from RCTs was likely to be scarce─ this was particularly with the aim of including all different types of tear and antibiotic regimens, in different settings. Studies investigating all types of perineal trauma were eligible, including first- and second-degree tears, episiotomies and OASIs. We included trauma sustained following spontaneous or assisted vaginal birth. All types of antibiotics at different doses, routes of delivery and duration were included. Non-English studies were translated. Studies were excluded where the antibiotics were used only therapeutically, where CRPT was not sustained, or where data was not available for our outcomes of interest. Case studies, case series and case controlled studies were excluded. A summary of correspondence with study authors is included in Table A in S1 Appendix, and a table of excluded studies in Table B in S1 Appendix.

### 2.3 Outcomes

The primary outcome was perineal infection. Secondary outcomes included: wound dehiscence, pain, wound healing, urinary incontinence, faecal incontinence, dyspareunia, length of hospital stay, hospital readmission and maternal adverse reaction. Outcomes were defined as per the reports of the study author and where available these definitions were included in the study characteristics table. There is not an existing core outcome set for CRPT and therefore the list of outcomes chosen from outset were based on author consensus and from prior discussions with the CHAPTER patient advisory group.

### 2.4 Search strategy

A systematic literature search was undertaken using: Medline, Embase, CINAHL, Cochrane central register of controlled trials, Web of Science and Google Scholar, from inception to February 2024. A combination of medical subject headings (MeSH) and text words were used. An example of search terms used is included in Appendix A in S1 Appendix, which were adapted for each database searched.

### 2.5 Study selection and data extraction

Two reviewers independently reviewed titles and abstracts of the retrieved articles (HA, RM), with a third independent reviewer resolving any conflicts (VHM). This process was repeated for full text screening and data extraction. Covidence software was used for study screening. Data from the studies meeting the inclusion criteria was extracted onto a data entry form and then transferred onto an electronic spreadsheet.

### 2.6 Risk of bias assessment

Two review authors assessed risk of bias for each study (HA, RM) with conflicts solved by consensus. Risk of bias was assessed in line with guidance from the Cochrane Handbook for Systematic Reviews of Interventions, using the RoB2 tool for RCTs and ROBINS-I tool for observational studies [16,17]. Both tools were applied at the outcome level as per guidance [16,18,19].

### 2.7 Certainty of evidence

The GRADE approach [20] was used to assess the certainty of the included evidence from the meta-analyses based on RCTs only─ these analyses were deemed most appropriate to apply the GRADE framework to as the effectiveness of an intervention was being considered.

### 2.8 Data analysis

Data was synthesised using Review Manager 5.4 software. To assess dichotomous outcomes, risk ratios (RR) were calculated, with 95% confidence intervals. An absolute risk reduction (ARR) and number needed to treat (NNT) were calculated using standard formulas for the primary outcome [21]. For continuous outcomes, means and standard deviations were extracted. A random effects model was applied, as studies were conducted using different antibiotic protocols amongst different populations and at different time-points, so were likely to be estimating different underlying effects. To assess for statistical heterogeneity the I^2^ statistic was used. Where data was not available in primary studies, no assumptions or imputations were made and only available data was used for meta-analysis. Where there were three or more RCTs eligible for inclusion, we performed an RCT only analysis in addition to an overall analysis pooling both observational studies and RCTs. Subgroup analyses were planned for outcomes where meta-analysis was possible; by degree of tear, route of antibiotic administration, risk of bias judgement, type of antibiotic received and spontaneous/assisted vaginal birth.

### 2.9 Publication bias

Funnel plots were generated to assess for publication bias in meta-analyses. Egger’s regression test was used to quantitavely assess for funnel plot asymmetry where there were ≥10 studies in the analysis [22]. Funnel plots and Egger’s regression test were generated using R software (4.3.0) and RStudio.

## 3 Results

### 3.1 Included studies

**Fig 1:**
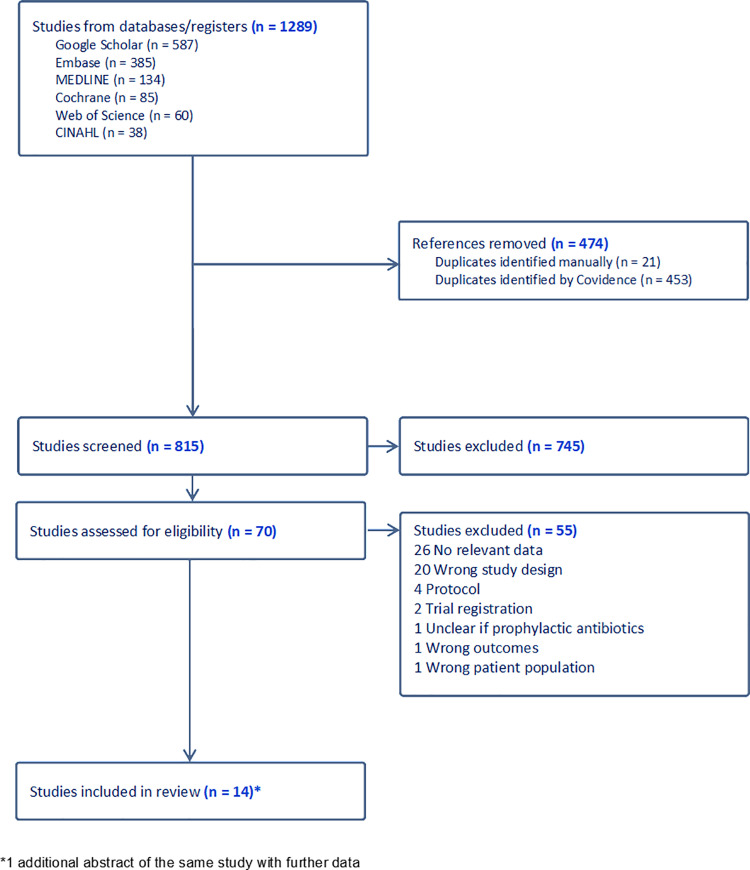
Study flow diagram. Study flow diagram illustrating the search and selection process generated using Covidence software (aligned to PRISMA 2020).

Fourteen full-text studies were eligible for inclusion, which comprised eight RCTs and six observational studies (two prospective cohort studies, one retrospective cross sectional study, one prospective cross-sectional study, one quality improvement initiative [retrospective data collection], one cohort study with retrospective and prospective components), involving 8,878 women across nine countries [11,23–35](Fig 1). Four studies were conducted in the USA, three in India and one in each of; Iran, UK, Brazil, South Africa, Germany, Thailand and Turkey. Characteristics of included studies can be found in Table 1. We included data from one additional published abstract from the full-text by Propst et al., as it contained further outcome data not included in the full-text version [36]. Humphrey’s et al., a secondary analysis of the ANODE (Antibiotics in the Prevention of Infection after Operative Vaginal Delivery) RCT by Knight et al., was included, as we were able to derive the total number with perineal trauma from Humphreys et al., and the number with perineal wound infection from the primary ANODE trial [10,27]. Where Humphreys is cited in results, this refers to results from both published articles.

**Table 1:** Characteristics of included studies. Table Abbreviations: RCT = randomised controlled trial, IV = intravenous, GDM = gestational diabetes mellitus, PIH = pregnancy induced hypertension, PROM = premature rupture of membranes, REEDA = redness, edema, ecchymosis, discharge and approximation of wound edges, OASI = obstetric anal sphincter injury, HIV = human immunodeficiency virus, IBD = inflammatory bowel disease, PPROM = preterm premature rupture of membranes, PPH = postpartum haemorrhage, DM = diabetes mellitus, Hb = haemoglobin, HTN = hypertension, VAS = visual analogue scale.

Author and year	Country	Study design, funding information	Population	Total number of participants in analysis	Antibiotic information	Eligibility criteria	Maternal outcomes, definitions and time at which wound infection/dehiscence checked for
Chandrababu 2019 [23]	India	RCT Funding: not reported	1^st^/2^nd^ degree tears Episiotomy Spontaneous/operative vaginal delivery	300	Split into A (IV) and B (oral) groups: A: Augmentin (clavulanic acid 200mg + amoxicillin 1000mg) 1.2 g IV single dose received just before episiotomy B: Augmentin (clavulanic acid 125mg + amoxicillin 500mg) 625 mg oral twice daily for 3 days after delivery	Full-term normal delivery Exclusion: patients with associated comorbidities – anaemia, GDM, PIH, PROM, patients allergic to the antibiotics used, patients with leukocyte counts >25,000	• Wound dehiscence = wound gape • Wound healing = mean REEDA score used at time of discharge • Time at which checked for wound dehiscence unreported
Cox 2021 [30]	USA	Quality improvement initiative, Funding: National Institute of Child Health and Human Development (NICHD)	3^rd^/4^th^ degree tears Spontaneous or operative vaginal delivery	512	Cefazolin 2g IV plus metronidazole (IV or oral) or clindamycin 900mg plus gentamicin 5mg/kg if penicillin allergic (recommended antibiotics however a minority of women received other antibiotic regimens) At time of OASI repair	All deliveries complicated by OASI for included study time period Exclusion criteria: none	• Perineal infection = ‘wound infection’ or ‘wound breakdown’ included from medical records • Infection checked for within 3 months of delivery
Duggal 2008 [11]	USA	RCT Funding: not reported	3^rd^/4^th^ degree tears Including spontaneous/operative vaginal delivery	107	Cefotetan or cefoxitin 1g IV or clindamycin 900mg IV if penicillin allergic Single dose at time of repair	Exclusion: patients <18 years old, group B strep positive, HIV positive, chorioamnionitis or history of IBD, already on antibiotics for any indication	• Perineal infection = perineal wound complication – purulent discharge or abscess and breakdown of repair site • Wound dehiscence = wound disruption • Infection checked at discharge and 2 weeks postpartum
Garala 2019 [24]	India	RCT Funding: no funding sources	1^st^/2^nd^ degree tears Episiotomy Spontaneous vaginal delivery only	146	Amoxicillin 500mg orally three times a day for 5 days Commenced after normal delivery	Spontaneous or induced labour, term or preterm without risk factors/complication Exclusion: PROM, PPROM, other infections/antibiotic use in 2 weeks before delivery, patients with cardiac disease, diabetes, GDM, immunosuppressant drug usage, prolonged labour, retained placenta, PPH	• Perineal infection = episiotomy wound infection - redness/excessive swelling, throbbing pain or tenderness, red streaks, pus or watery discharge, chills or fever • Length of hospital stay • Checked for infection at 6 weeks postpartum
Gommesen 2019 [25]	Germany	Prospective cohort Funding: Odense University hospitals research foundation, The Region of Southern Denmark, University of Southern Denmark, the department of Gynecology and Obstetrics, Odense University hospital, A.P. Møller and wife Chastine Mc-Kinney Møllers foundation and The Danish Association of Midwives	1^st^/2^nd^/3^rd^/4^th^ degree tears Episiotomy Spontaneous or operative vaginal delivery	390	Antibiotic data unreported. Timing started during birth/postpartum period.	Primiparous women > 18 years old, able to read and speak Danish Exclusion: not reported	• Perineal infection = episiotomy site infection - purulent drainage or wound abscess • Wound dehiscence = a gap between wound edges >0.5 cm • Infection checked for at 11–21 days postpartum
Goodarzi 2020 [26]	Iran	RCT Funding: not reported	Episiotomy only Spontaneous vaginal delivery only	140	Cephalexin 500mg oral every 6 hours for 7 days Commenced after episiotomy	Primiparous women Exclusion: not reported	• Wound dehiscence = non-approximation of wound edges • Wound healing = REEDA score at 7 days • Hospital readmission • Wound healing assessment 7 days after birth
Humphreys 2022 [27]	UK	Secondary analysis of RCT (also required information from the primary RCT Knight 2019 ^9^) Funding: The Knight 2019 trial was funded by the National Institute for Health Research (NIHR) Health Technology Assessment Programme	1^st^/2^nd^ degree tears Episiotomy Operative vaginal delivery only	3147	Co-amoxiclav – 1g amoxicillin, 200mg clavulanic acid, IV single dose Started as soon as possible after birth and within 6 hours	Forceps/vacuum-assisted birth at > 36 weeks and > 16 years old Exclusion: indication for ongoing prescription of antibiotics in postpartum period (e.g., 3rd/4^th^ degree tear), contraindications to prophylactic amoxicillin and clavulanic acid	• Perineal infection = a new prescription of antibiotics for presumed perineal wound-related infection. • Checked for infection within 6 weeks of delivery
Lewicky-Gaupp 2015 [31]	USA	Prospective cohort Funding: not reported	3^rd^/4^th^ degree tears Spontaneous or operative vaginal delivery	268	Cefazolin or cephalexin IV single dose or/and oral amoxicillin or amoxicillin and clavulanate or metronidazole or clindamycin or cephalexin or penicillin for 7 days Dose unreported Started at time of repair	Full term deliveries complicated by OASI Exclusion: multifetal pregnancies, premature < 37 week delivery	• Perineal infection = perineal complication – three or more of heat, erythema, purulent discharge, or wound breakdown- at least 1 cm, or both • Checked for complications within 1 week of delivery and at 2, 6, and 12 weeks post-birth
Neto 1990 [35]	Brazil	RCT Funding: not reported	Episiotomy	73	Chloramphenicol 500mg oral every 6 hours for 72 hours Started after repair	Normal birth with episiotomy with or without risk factors for development of puerperal infection	• Perineal infection = pain, heat, redness, purulent discharge and separation of the wound edges • Wound dehiscence = separation of wound edges without signs of infection • Reviewed for complications within 10 days of birth
Propst 2022 [32]	USA	Cohort - prospective and retrospective components (also using information from standalone conference abstract of the same study) [36] Funding: not reported	3^rd^/4^th^ degree tears Spontaneous or operative vaginal delivery	311	Cefazolin most commonly used. Also; cefoxitin, clindamycin, clindamycin and gentamicin, ampicillin, cefazolin and metronidazole, cefotetan, ciprofloxacin and metronidazole, ampicillin/sulbactam Dose and route unreported Started at time of delivery	OASI sustained at time of vaginal delivery and consented to research registry at Cleveland clinic Ohio Exclusion: < 18 years old	• Perineal infection = wound infection – at least three of erythema, oedema, warmth or purulent discharge • Wound dehiscence = wound breakdown - separation of layers deeper than the skin ≥1 cm • Unclear time point at which wound infection was recorded • Urinary incontinence- present/absent (used in systematic review) and Urinary Stress Inventory-6 • Faecal incontinence- present/absent (used in systematic review) and Faecal Incontinence Severity Index • Initial postpartum visit at 12 days postpartum (median) then 6–12 month follow-up
Sebitloane 2008 [28]	South Africa	RCT Funding: Supported by grant RES 112–02 from Secure the Future-HIV Research Institute (Bristol-Myers Squibb, New York, NY)	Episiotomy data only Spontaneous or operative vaginal delivery	195	Cefoxitin 2g IV single dose Started during active labour when cervical dilatation > 3 cm	HIV-infected women >18 years old Exclusion: HIV uninfected/didn’t want to know HIV status, planned or emergency caesarean delivery, allergic to penicillin	• Perineal infection = infected episiotomy (with presence of seropurulent/purulent exudate) • Infection checked for at 24–72 hours, 1 week and 2 weeks post-birth
Tandon 2018 [29]	India	RCT Funding: not reported	Episiotomy only Spontaneous vaginal delivery only	300	Cefixime 200mg twice daily and metronidazole 400mg three times a day orally for 5 days Time started unreported	Exclusion: extension of episiotomy as a 3^rd^/4^th^ degree injury, anaemia with Hb < 8, intrapartum antibiotics, induced labour, vaginal leaking > 24 hrs, systemic diseases – DM, jaundice, HTN, heart disease, chronic renal disease	• Perineal infection = any positive findings – redness/pain/swelling/wound discharge/gape • Wound dehiscence = wound gape • Wound healing = completely healed wounds at day 10 follow up • Pain = presence or absence of pain in the form of yes/no and further graded according to the VAS score for pain on scale 1–10 • Infection checked for within 5 days after birth
Thongtip 2023 [33]	Thailand	Retrospective cross-sectional Funding: not reported	All who underwent repair of perineal trauma; infer 1^st^/2^nd^/3^rd^/4^th^ degree tears and episiotomies. Spontaneous vaginal delivery only	2589	Cefoxitin 1g or 900mg clindamycin (if penicillin allergic) IV single dose for 3^rd^/4^th^ degree tears Oral clindamycin or amoxicillin for other indications Started after vaginal delivery	Exclusion: no perineal tear, gestational age < 28 weeks, birth weight <1000 grams, incomplete medical records	• Perineal infection = purulent discharge and positive bacterial culture • Wound dehiscence = gap of > 1 cm between wound edges • Infection within 72 hours post delivery
Yilmaz 2024 [34]	Turkey	Prospective cross-sectional Funding: reports no external funding	Episiotomy only Spontaneous vaginal delivery	400	Rifampicin 250mg in 3ml topical single dose Irrigated after episiotomy	Exclusion: patients with conditions affecting wound healing, episiotomies progressing to 3^rd^/4^th^ degree lacerations	• Perineal infection = episiotomy infection - presence of elevated temperature, severe local pain, purulent discharge, colour change from site of incision, wound dehiscence • Wound dehiscence = episiotomy dehiscence • Infection checked for at 1 week, 3 weeks and 1 month post delivery

Studies used varying prophylactic antibiotic regimens. The most commonly used antibiotics were cefoxitin (four studies), co-amoxiclav (three studies) and cefazolin (three studies). Antibiotics were commenced at varying time points─ as detailed in Table 1, most commonly antibiotics were administered at the time of repair or after birth. There were varying definitions of perineal infection reported in the included studies (detailed in Table 1). Wound breakdown was included in the definition of perineal infection in five studies. Purulent discharge was also one of the most common signs included in definitions of perineal wound infection. The presence of infection was recorded at different time-points, ranging from 24 hours to three months postpartum.

### 3.2 Risk of bias

#### 3.2.1 Randomised controlled trials.

Four studies were deemed to be at high risk of bias, two with some concerns and two low risk of bias. Fig A in S1 Appendix shows the overall quality of the RCTs split by the RoB2 tool domains. Domain two, bias due to deviations from intended interventions carried the highest risk of bias. There was a lack of blinding in several included RCTs.

#### 3.2.2 Observational.

All six observational studies were deemed to be at critical or serious risk of bias. Fig B in S1 Appendix shows the overall quality of the observational studies split by the ROBINS-I tool domains. The ROBINS-I risk of bias tool determines bias by comparison of the observational interventional study to a fully randomised trial. As such, in domain one (risk of bias due to confounding), a study can only be classified as low risk of bias if the study is comparable to a well-performed randomised trial and therefore no studies were deemed at low risk of bias in this domain. Furthermore, the majority of outcome data used in this systematic review from observational studies was inherently not controllable and therefore a critical risk of bias was assigned for the first domain. Authors made the decision to still include these studies, whilst performing an RCT only sensitivity analyses. Whilst some studies presented baseline characteristics between intervention and no intervention groups, others did not present this data at all or in full.

### 3.3 GRADE assessment

Table C in S1 Appendix shows the Summary of Findings table. Overall the GRADE certainty of findings were low for both perineal infection and wound dehiscence. Several studies included in each analysis had a high or unclear risk of bias using the RoB2 and ROBINS-I tools. There were differences in the populations and interventions between the included studies, with Sebitloane et al. [28] solely including a HIV cohort, affecting the directness of the evidence. Varying antibiotic types, doses, durations and routes were utilised between studies. Heterogeneity was present in the wound dehiscence analysis, with some point estimates demonstrating an effect in the opposite direction.

### 3.4 Data analysis

**Fig 2:**
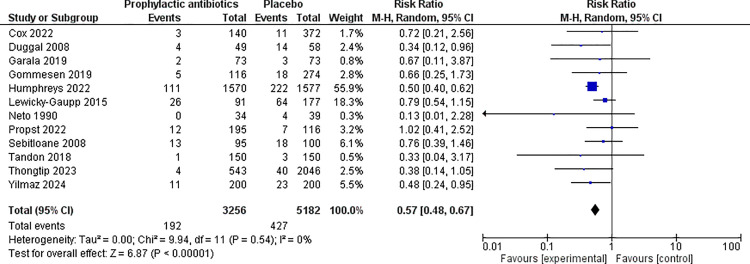
Perineal wound infection─ prophylactic antibiotics compared to none. Forest plot to show the outcome of perineal wound infection for prophylactic antibiotics compared to no prophylactic antibiotics. Created using Revman. Abbreviations M-H = Mantel-Haenszel, CI = confidence interval.

**Fig 3:**
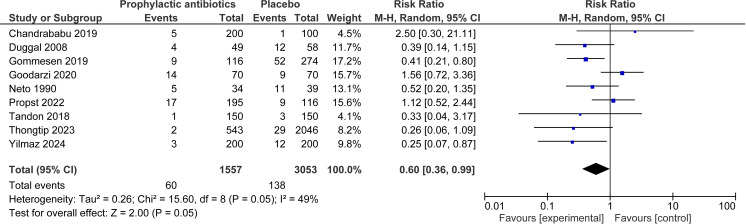
Perineal wound deshiscence─ prophylactic antibiotics compared to none. Forest plot to show the outcome of perineal wound dehiscence for prophylactic antibiotics compared to no prophylactic antibiotics. Created using Revman. Abbreviations M-H = Mantel-Haenszel, CI = confidence interval.

For perineal wound infection, when observational studies and RCTs were pooled together, 12 studies were included in the analysis, involving 8,438 women (Fig 2) [11,24,25,27–35]. Overall, there was association with reduced rates of infection in those administered prophylactic antibiotics (RR 0.57, 95% CI 0.48 to 0.67, I^2^ = 0%). For perineal wound dehiscence when RCTs and observational studies were pooled together, nine studies were included, with 4,610 women [11,23,25,26,29,32–35]. Overall, there was an association with reduction in wound dehiscence events in the prophylactic antibiotic group compared to the placebo group (RR 0.60, 95% CI 0.36 to 0.99, I^2^ = 49%) (Fig 3).

Data collected for the remaining secondary outcomes was solely descriptive as there was insufficient information for meta-analysis. Chandrababu et al. and Goodzari et al. measured wound healing using the REEDA (redness, oedema, ecchymosis and approximation) wound assessment tool, which scores each parameter on a scale of zero to three, with a higher score representing poorer wound healing and a total score from 15 subsequently calculated [23,26]. In the former study there was no statistically significant difference in the rate of wound healing between those receiving compared to not receiving antibiotics, when measured at the time of discharge [23]. Goodarzi et al. reported a mean REEDA score of 1.14 in the prophylactic antibiotics group versus 1.63 in the control group [26].

Tandon et al. reported the outcome of pain using both a visual analogue scale and in terms of the presence or absence of pain [29]. There was no significant difference in the presence or absence of pain between antibiotic and no antibiotic groups (p = 0.99), but there was a statistically significant difference between groups when pain was recorded using a visual analogue scale (p = 0.026).

The abstract by Propst et al. was the only study to report data for urinary and faecal incontinence [36]. It was reported immediately and at six to 12 months postpartum. There was no statistically significant difference in urinary or faecal incontinence between those receiving antibiotics versus no antibiotics immediately postpartum. This was consistent for urinary and faecal incontinence at six to 12 months postpartum where no significant differences were found [36]. For the outcome of length of hospital stay, Garala et al. found no statistically significant difference in those receiving compared to not receiving prophylactic antibiotics [24]. Goodarzi et al. found two cases of hospital readmission in the prophylactic antibiotics arm compared to none in the control arm (n = 70 in each arm) [26]. Goodarzi et al. was the only study to comment on maternal adverse reactions to antibiotics and there were no observed adverse reactions reported [26]. No included studies reported on dyspareunia.

### 3.5 Sensitivity and subgroup analyses

#### 3.5.1 Sensitivity analyses.

**Fig 4:**
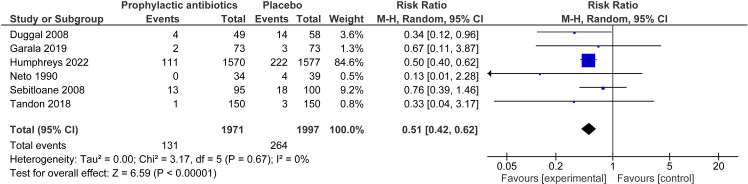
Perineal wound infection─ prophylactic antibiotics compared to none (RCT only analysis). Forest plot to show the outcome of perineal infection for prophylactic antibiotics compared to no prophylactic antibiotics for RCTs only. Created using Revman. Abbreviations M-H = Mantel-Haenszel, CI = confidence interval.

**Fig 5:**
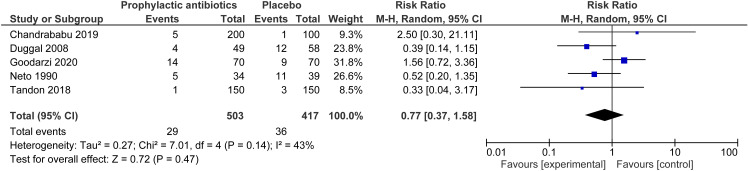
Perineal wound deshiscence─ prophylactic antibiotics compared to none (RCT only analysis). Forest plot to show the outcome of wound dehiscence for prophylactic antibiotics compared to no prophylactic antibiotics for RCTs only. Created using Revman. Abbreviations M-H = Mantel-Haenszel, CI = confidence interval.

The RCT only analysis for the outcome of perineal wound infection included six studies involving 3,968 women [11,24,27–29,35]. This showed a significant reduction in the risk of perineal infection with prophylactic antibiotics, across all types of tear (RR 0.51, 95% CI 0.42 to 0.62, I^2^ = 0%) (Fig 4). Calculation of the absolute risk for the RCT only analysis gave a reduction of 66 perineal infection events per 1000 women when prophylactic antibiotics were given compared to no prophylactic antibiotics. The NNT was 15. For the perineal wound dehiscence RCT only analysis, including five studies and 920 women, there was no statistically significant difference between risk in the prophylactic antibiotic versus placebo group (RR 0.77, 95% CI 0.37 to 1.58, I^2^ = 43%) (Fig 5) [11,23,26,29,35].

#### 3.5.2 Subgroup analyses.

**Fig 6:**
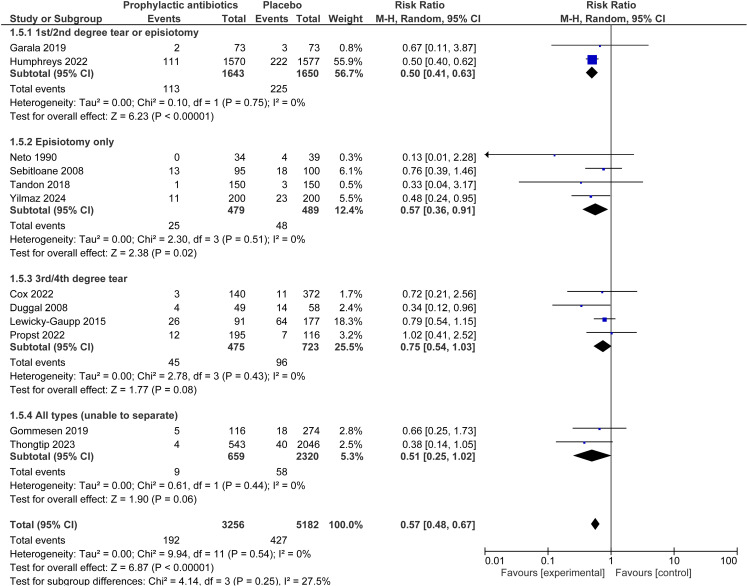
Perineal wound infection subgrouped by type of perineal trauma─ prophylactic antibiotics compared to none. Forest plot to show the outcome of wound infection for prophylactic antibiotics compared to control where subgroups are split by type of perineal tear. Created using Revman. Abbreviations M-H = Mantel-Haenszel, CI = confidence interval.

For the outcomes of infection and dehiscence in those receiving prophylactic antibiotics compared to no antibiotics, subgroup analysis was performed by type of tear, across observational studies and RCTs. For the outcome of infection in the first/second degree tear or episiotomy, episiotomy only, third/fourth degree tear and all types of perineal trauma (unable to separate by tear type) subgroups, results were RR 0.50, 95% CI 0.41–0.63, I^2^ = 0%; RR 0.57, 95% CI 0.36–0.91, I^2^ = 0%; RR 0.75, 95% CI 0.54–1.03, I^2^ = 0% and RR 0.51, 95% CI 0.25–1.02, I^2^ = 0% respectively (p = 0.25 for subgroup differences) (Fig 6). For the outcome of wound dehiscence, in the same tear subgroups respectively, results were RR 1.64, 95% CI 0.80–3.39, I^2^ = 0%; RR 0.39, 95% CI 0.19–0.80, I^2^ = 0%; RR 0.71, 95% CI 0.26–1.97, I^2^ = 59%; RR 0.38, 95% CI 0.20–0.69, I^2^ = 0% (p = 0.01 for subgroup differences) (Fig C in S1 Appendix).

We performed subgroup analyses investigating the effect of the route of administration of antibiotics on risk of perineal infection. There were two main routes of administration used in the included studies; intravenous and oral, and one study by Yilmaz et al. used a topical route [34]. Some studies administered antibiotics via a mixture of routes and others did not specify. For perineal wound infection, results for studies administering antibiotics intravenously, orally, topically or via mixed/unspecified routes were RR 0.52, 95% CI 0.43–0.63, I^2^ = 0%; RR 0.39, 95% CI 0.11–1.38, I^2^ = 0%; RR 0.48, 95% CI 0.24–0.95 [I^2^ not applicable]; RR 0.75, 95% CI 0.55–1.02, I^2^ = 0% respectively (p = 0.24 for subgroup differences) (Fig D in S1 Appendix). For perineal wound dehiscence, results for the same subgroups respectively were RR 0.39, 95% CI 0.14–1.15 [I^2^ not applicable]; RR 0.82, 95% CI 0.33–2.05, I^2^ = 49%; RR 0.25, 95% CI 0.07–0.87 [I^2^ not applicable] and RR 0.64, 95% CI 0.29–1.44, I^2^ = 56% (p = 0.43 for subgroup differences) (Fig E in S1 Appendix).

Subgroup analyses by risk of bias category were also performed to investigate whether the effect of antibiotics on perineal wound infection or wound dehiscence was altered by study quality. For perineal wound infection subgrouped by studies at high/critical/serious, some concerns and low risk of bias, results were RR 0.67, 95% CI 0.51–0.89, I^2^ = 0%; RR 0.76, 95% CI 0.39–1.46, I^2^ = N/A; RR 0.49, 95% CI 0.40–0.61, I^2^ = 0% (p = 0.14 for subgroup differences) (Fig F in S1 Appendix). For perineal wound dehiscence, for the same bias categories respectively, results were RR 0.53, 95% CI 0.31–0.88, I^2^ = 32%; RR 1.56, 95% CI 0.72–3.36, I^2^ = N/A; RR 0.39, 95% CI 0.14–1.15, I^2^ = N/A (p = 0.04 for subgroup differences) (Fig G in S1 Appendix).

Meta-analysis by type of antibiotic used was not possible as there were not sufficient studies using the same antibiotic. Many of the observational studies used several different types of antibiotics within their single dataset and it was not possible to separate the outcome data by antibiotic used. Type of antibiotic used in each study can be seen in Table 1. Similarly subgroup analysis by spontaneous/assisted vaginal birth was not possible, as there was insufficient data for analysis.

### 3.6 Publication bias

Funnel plots, generated for outcomes of infection and wound dehiscence can be found in Fig H and Fig I in S1 Appendix. Funnel plots suggest an absence of significant publication bias.

## 4 Discussion

### 4.1 Main findings

There is a reduction in perineal wound infection when prophylactic antibiotics are received, compared to placebo (absolute risk reduction 6.6% based on the RCT only analysis) across all types of tear. Subgroup analysis demonstrated a reduced risk of infection when prophylactic antibiotics were administered in the non-OASI groups. For the prevention of wound dehiscence, prophylactic antibiotics were not found to be superior to placebo or control in our RCT only analysis.

### 4.2 Context within previous research

The 2019 ANODE trial has provided clear evidence that antibiotics for assisted vaginal births are effective in preventing subsequent infections and therefore this has now been incorporated into guidelines and clinical practice [10]. Whilst our systematic review includes data from the ANODE trial, we also include studies where women underwent a spontaneous vaginal birth. Our results, whilst incorporating data from women with all types of CRPT and across all modes of vaginal birth and therefore not being directly comparable to ANODE, were overall in keeping with this robust and recent RCT.

In terms of existing systematic reviews on the subject, the Cochrane review investigating prophylactic antibiotics for episiotomy repair was published in 2017 [13]. Our systematic review includes the Neto 1990 RCT, which is the only trial included in the 2017 Cochrane review [35]. Based on the findings of the single, small RCT, the 2017 Cochrane review found that there was no clear difference between the groups who received antibiotics compared to those who did not. This trial is now 35-years-old and authors of the primary RCT used chloramphenicol as their prophylactic antibiotic─ this is clearly not an antibiotic that would be considered for this use in present settings. In our systematic review, we found a greater number of studies investigating the use of prophylactic antibiotics after episiotomy and therefore can build on evidence from this earlier Cochrane review. Furthermore, our subgroup analyses by type of tear suggested that prophylactic antibiotics for women who undergo episiotomy may be associated with a reduction in rates of wound infection and wound dehiscence. A further Cochrane review investigated prophylactic antibiotics for third and fourth degree tears and found one eligible RCT─ this trial suggested that prophylactic antibiotics were effective in preventing wound infection after OASI [11,37]. We found no additional RCTs focusing solely on women with OASI, however we were also able to draw on studies with observational design.

Our review must also be considered in the context of the recently published UK governmental Birth Trauma report, which highlights poor postnatal care as a significant contributor to trauma [9]. The devastating effects of perineal trauma are discussed in this report, stressing the urgency with which evidence-based improvements to care must be made. Our review therefore begins to unpick the existing evidence behind a simple intervention that may help to improve the lives of many women and their families.

### 4.3 Strengths and limitations

To our knowledge there is no existing up-to-date systematic review and meta-analysis which considers the use of prophylactic antibiotics amongst all types of CRPT. This work therefore begins to fill a notable gap in the evidence base, whilst additionally highlighting where further primary research is needed. A broad range of search terms were applied in relevant databases, producing a comprehensive analysis. The inclusion of non-English language studies and grey literature increases the number of studies on which conclusions can be drawn and improves the generalisability of our results. This review includes a larger number of studies in comparison to existing systematic reviews, meaning that our results provide increased breadth and depth to the subject area.

Notably, 10 of 14 included primary studies were determined to have a high, critical or serious risk of bias, which means our conclusions are based on studies which provide overall, less robust evidence. Whilst it is a strength that we included studies conducted across nine different countries, we found no studies eligible for inclusion conducted in low-income countries. This highlights a significant gap in the evidence base and we must ensure that future research considers the applicability and effectiveness of interventions in different healthcare settings.

Sebitloane et al. includes a HIV cohort, highlighting the use of antibiotics in particular groups who may be more likely to experience complications [28]. Inclusion of this trial must be considered when drawing conclusions, as these results may not be generalisable to the wider population. Many of the secondary outcomes were patient reported outcomes, with few eligible studies reporting them and therefore, due to scarcity of data, meta-analysis was not possible. These subjective outcomes were also increasingly likely to have been recorded and reported differently between studies. Additionally, the definitions of outcomes varied considerably across studies, with a lack of consistency in the definition of wound infection (see Table 1). The pooling of various antibiotic regimens used in the included studies, increases heterogeneity within our results, an additional limitation to this review.

A further important limitation to our systematic review is that whilst the inclusion of observational studies increased the available data for meta-analysis, only association can be demonstrated in these studies. We attempted to mitigate this by also performing analysis limited to RCTs only.

It is also important to note that due to both lack of reporting in primary studies and the inclusion of women who had undergone both assisted and spontaneous vaginal birth in the same study populations, we were not able to perform subgroup analysis by birth mode. As currently in the UK, women who undergo an assisted vaginal birth receive prophylactic antibiotics, it is important that we can ascertain the benefit of prophylactic antibiotics in those who experience spontaneous vaginal birth if an effect is truly there. Subgroup analysis by type of perineal trauma was also limited by reporting in primary studies, as the majority of studies included women with several different tear types (see Table 1) and results for each distinct type of tear were not completely separable. This therefore means that our subgroup analysis by perineal trauma type is restricted to more broad groups of perineal trauma and we are not able to draw any more granular conclusions.

### 4.4 Clinical implications and future research

Whilst our review demonstrates a reduction in perineal wound infections after antibiotics are received, when pooling data from all types of tear and from those with both assisted and normal vaginal births, there was scarce reporting of any potential harms in the included studies. In any future trials, we must ensure that consideration is given to minimising antibiotic excretion into breastmilk, through careful selection of the antibiotic type and duration of exposure. In terms of long-term risks to the infant, a recent large data-linkage study found no evidence of an increase in long-term health conditions such as asthma, eczma, autoimmune disease or neurodevelopmental issues in infants exposed to prophylactic antibiotics, administered prior to incision at caesarean section [38]. Whilst the data-linkage study investigated antibiotics given antenatally, prophylactic antibiotics for perineal trauma would likely result in less exposure to the infant, as administration would be postnatally.

One of the reasons it is hard to draw firm conclusions from our systematic review, is that a wide range of antibiotics were used, in some cases within the same study. Potential options for further investigation in a future trial are co-amoxiclav and the cephalosporin class of antibiotics. Both of these represent penicillin based antibiotics which can be associated with increasing resistance and Clostridium difficile infection and therefore this must be considered when balancing the risks and benefits [39].

There is also a need to consider the use of non-antibiotic interventions for reducing the risk of infection after perineal trauma. This includes maintaining a sterile field during repair of perineal trauma and ensuring sterile drapes and cleaning solution are utilised for repairs in every setting. Other interventions such as use of sanitary pads with antibacterial properties, require further investigation [40]. Prior to performing a further RCT, there is a need for development of a CRPT core outcome set, in order to ensure the outcomes captured are those most important to women and clinicians. Particularly notable in this review were the lack of widely used and validated wound assessment tools for healing of perineal trauma and the lack of standardised patient reported outcome measures. The wider CHAPTER project aims to develop these, so they are available for future trials. Additionally, further research should work to identify the characteristics of women at higher risk of CRPT complications, who would subsequently be likely to obtain the most benefit from intervention.

## 5 Conclusion

Although our meta-analysis demonstrates that prophylactic antibiotics significantly reduce the risk of perineal infection, there are not sufficient high-quality RCTs to adequately inform guideline change amongst women with first/second degree tears or episiotomies. The lack of consistency across primary research studies on the definition of perineal wound infection and the unavailability of outcome data for distinct tear types were important limitations to this review. Our findings highlight the need for further research, with a robust and sufficiently powered RCT amongst women with spontaneous vaginal births and non-OASI perineal trauma, required to provide a definitive answer. Any further trial involving antibiotics must also capture any potential maternal and infant harms from antibiotic administration and must consider the implications at a wider population level. As is evident from the recent Birth Trauma Report, improving postnatal care is imperative to improving women’s experiences of childbirth─ we must therefore ensure that any potential interventions to reduce complication rates after CRPT are fully investigated and that this remains a priority for researchers and clinicians.

## Supporting information

S1 Appendix Author correspondence, search terms, excluded studies, risk of bias, additional analyses. (DOCX)

S2 Appendix Additional information. (XLSM)

S3 Appendix Citations retrieved in searches. (XLSX)

S4 Appendix PRISMA checklist. (DOCX)
