# Supplementary material for: Antibiotic prophylaxis for childbirth-related perineal trauma: A systematic review and meta-analysis
Source: PLoS One. 2025 May 9;20(5):e0323267. doi: 10.1371/journal.pone.0323267 (PMC12064200; doi:10.1371/journal.pone.0323267)
Supplement: S1 Appendix — (DOCX) [file pone.0323267.s001.docx]

Table A: Summary of email correspondence with primary study authors

| Study corresponding author emailed | Reason for email | Date of reply | Response | Our response |
| --- | --- | --- | --- | --- |
| Cox 2021 | To obtain separate data as a composite of wound infection and breakdown was reported | - | None | Included in analysis of infection (excluded from RCT sensitivity analysis due to study design) |
| Feng 2021 | To check if indication for antibiotic administration was prophylactic or not | - | None | Study excluded as case control |
| Gommesen 2019 | To check if indication for antibiotic administration was prophylactic or not | 20/2/24 | Replied – unable to confirm reasoning participants received antibiotics | Discussed amongst authors. Included in analysis (excluded from RCT sensitivity analysis due to study design) |
| Humphreys 2022/Knight 2019 | To check whether there was any double counting between the subgroups of types of perineal infection | 1/3/24 | Replied –confirmed no double counting between different infection subtypes | Included |
| Lewicky-Gaupp 2015 | To obtain separate data as a composite of wound infection and breakdown was reported | - | None | Included (excluded from RCT sensitivity analysis due to study design) |
| Propst 2022 | To check if participants in abstract and manuscript were the same | 19/2/24 | Replied- some repeats but no quantification could be made | Both abstract and manuscript included as they measured different outcomes so there was no double counting |

Table B: Table of studies obtained for full-text screening with reasons for exclusion if applicable

| **Author and year** | Country | Study design | Population | Patients | Exclusion reason |
| --- | --- | --- | --- | --- | --- |
| **Excluded studies** | | | | | |
| 1. Bachmeier 2019 | Canada | Presentation | - | - | No relevant data |
| 1. Banu 2020 | Japan | Observational | Ob-Gyn surgery | 113 cases | No relevant data |
| 1. Barg 2023 | Israel | Multicentre national survey | All births | 164 completed surveys | No relevant data |
| 1. Blaser 2020 | USA | Review | - | - | Review/editorial |
| 1. Buppasiri 2014 | UK - Cochrane | Systematic review | 3^rd^ and 4^th^ degree tears | 147 women | Systematic review |
| 1. Calvia 2016 | UK | Audit | Perineal tear | 66 patients | No relevant data |
| 1. Cawich 2008 | Jamaica | Case series | 3^rd^/4^th^ degree tears  All vaginal deliveries | 5 | Case series |
| 1. Cherian 2022 | India | RCT | Operative vaginal delivery  Checked for infection within 6 weeks delivery | 700 women | Trial registration |
| 1. Duffy 2019 | USA | Observational | 3^rd^/4^th^ degree laceration | 92,320 women | No relevant data |
| 1. Dunk 2021 | USA | Retrospective cohort | Spontaneous or operative vaginal delivery | 14,646 deliveries | No relevant data |
| 1. Dwyer 2020 | America | Retrospective cohort | 3^rd^/4^th^ degree tear or episiotomy | 58,280 births | No relevant data |
| 1. Eschenbach 1980 | USA | Review | - | - | Review/editorial |
| 1. Eubanks 2017 | USA | Review | - | - | Review/editorial |
| 1. Feng 2021 | China | Case control | Forceps delivery  Poor versus normal wound healing | 148 women | Case control |
| 1. Fernandez 1993 | France | RCT | Episiotomy  Spontaneous or operative vaginal delivery | 610 | No relevant data |
| 1. Fiore 2004 | Europe | Case-control | Vaginal and elective caesarean deliveries | - | No relevant data |
| 1. Giugale 2017 | USA | Retrospective cohort | 3^rd^/4^th^ degree tears | 1708 women | No relevant data |
| 1. Gong 2021 | Canada | Cross-sectional | All types of perineal tear | 73 women | No relevant data |
| 1. Hibbard 1978 | USA | Observational | Recto-vaginal fistulas and complete perineal tears | 51 women | Wrong patient population |
| 1. Hughes 2022 | UK | Retrospective review & sequential prospective audits | Assisted deliveries | 34 deliveries | No relevant data |
| 1. Iwabu 2020 | Switzerland | Review | - | - | Review/editorial |
| 1. Knight 2018 | UK | RCT | Operative vaginal delivery  Checked for infection within 6 weeks delivery | - | Protocol |
| 1. Knight 2018 | UK | Protocol - ANODE | - | - | Protocol |
| 1. Knight 2019 | UK | Abstract – ANODE trial | Operative vaginal delivery | 3,420 women | Abstract |
| 1. Knight 2019 | UK | Review | - | - | Review/editorial |
| 1. Liabsuetrakul 2014 | Africa & Latin America | WHO global survey | Vaginal delivery, used antibiotics | 205 602 women | No relevant data |
| 1. Lian 2022 | China | Cross-sectional | - | 72,519 deliveries | Wrong outcomes |
| 1. Menzlova 2013 | Czech Republic | Review | - | - | Review/editorial |
| 1. Monga 1993 | USA | Article | - | - | No relevant data |
| 1. Montif 2008 | USA | Review | - | - | Review/editorial |
| 1. Munyao 2016 | Kenya | Observational | Episiotomy  Primiparous women | 174 participants | No relevant data |
| 1. Ngoc 2005 | Vietnam | Prospective cohort | - | 19,000 deliveries | No relevant data |
| 1. Okeahialam 2021 | UK | Review | - | - | Review/editorial |
| 1. Owens 2020 | USA | Cost effectiveness model | - | - | No relevant data |
| 1. Owino 2015 | UK | Protocol - ANODE | - | - | Protocol |
| 1. Paulsen 2019 | South Africa | Retrospective chart review | Individuals who had OASI repair | 85 participants | Dissertation |
| 1. Puri 2022 | India | Quality improvement initiative | - | - | No relevant data |
| 1. Rajabian - 2019 | Iran | RCT | Episiotomy only, primiparous women  Episiotomy site infection checked for at 7 days post delivery | 170 | Uncompleted clinical trial protocol |
| 1. Sagi-Dain 2020 | UK | Review | - | - | Review/editorial |
| 1. Setiawan 2022 | Indonesia | Literature review | - | - | Review/editorial |
| 1. Sharma 2021 | India | Quality improvement initiative | Low risk uncomplicated vaginal deliveries | - | No relevant data |
| 1. Sirilak 2022 | Thailand | Prospective Cohort | Episiotomy  Spontaneous vaginal delivery only  1^st^/2^nd^/3^rd^/4^th^ degree tears | 117 women | No relevant data |
| 1. Soliman 2023 | USA | Review | - | - | Review/editorial |
| 1. Stock 2013 | USA | Retrospective chart review | OASIs | 1629 women had OASIs | Unclear is prophylactic antibiotics |
| 1. Swidan 2021 | Egypt | RCT | Singleton pregnancies,  Artificial rupture of membrane during active phase of labour | - | No relevant data |
| 1. Tharpe 2008 | USA | Review | - | - | Review/editorial |
| 1. Thompson 2020 | Australia/UK | Review | - | - | Review/editorial |
| 1. Unknown | Unknown | Trial registration | Unknown | Unknown | Trial registration |
| 1. vanSchalkwyk 2010 | Canada | Review | - | - | Review/editorial |
| 1. Venkatesh 2019 | USA | Retrospective cohort | Singleton deliveries >23 wks gestation | 221, 274 deliveries | No relevant data |
| 1. WHO 2021 | Worldwide | Guidelines | - | - | Guideline/recommendation |
| 1. Woodd 2021 | Tanzania | Observational cohort | - | 879 women | No relevant data |
| 1. Yan 2022 | Laos | Observational study | - | 1,777 women | No relevant data |
| 1. Yang 2006 | China | Logistic regression analysis | Perineal lateral incision with and without incision infection | 315 cases | No relevant data |
| 1. Zimmerman 2020 | Switzerland | Review | - | - | Review/editorial |
| **Included studies** | | | | | |
| 1. Chandrababu | India | RCT | 1st/2nd degree tears  Episiotomy  Spontaneous/operative vaginal delivery | 300 women |  |
| 1. Cox 2021 | USA | Quality improvement | 3^rd^/4^th^ degree tears  Spontaneous or operative vaginal delivery | 512 women |  |
| 1. Duggal 2008 | USA | RCT | 3^rd^/4^th^ degree tears  Including spontaneous/operative vaginal delivery | 107 women |  |
| 1. Garala 2019 | India | RCT | 1^st^/2^nd^ degree tears  Episiotomy  Spontaneous vaginal delivery only  Checked for infection at 6 weeks postpartum | 146 women |  |
| 1. Gommesen 2019 | Germany | Prospective cohort | 1^st^/2^nd^/3^rd^/4^th^ degree tears  Episiotomy  Spontaneous or operative vaginal delivery  Infection checked for at 11-21 days postpartum | 390 women |  |
| 1. Goodarzi 2020 | Iran | RCT | Episiotomy only  Spontaneous vaginal delivery only  Checked for infection 7 days post delivery | 140 women |  |
| 1. Humphreys 2022 | UK | Secondary analysis of RCT | 1^st^/2^nd^ degree tears  Episiotomy  Operative vaginal delivery only  Checked for infection within 6 weeks of delivery | 3147 women |  |
| 1. Lewicky-Gaupp 2015 | USA | Prospective cohort | 3^rd^/4^th^ degree tears  Spontaneous or operative vaginal delivery  Infection checked for at discharge and 2 weeks postpartum | 268 women |  |
| 1. Neto 1990 | Brazil | RCT | Episiotomy | 73 women |  |
| 1. Propst 2022 | USA | Cohort - prospective and retrospective components | 3^rd^/4^th^ degree tears  Spontaneous or operative vaginal delivery  Unclear time point at which infection was recorded | 311 women |  |
| 1. Sebitloane 2008 | South Africa | RCT | Episiotomy data only  Spontaneous or operative vaginal delivery  Infection checked for at 24-72 hours post-birth and 1-2 weeks postpartum | 195 women |  |
| 1. Tandon 2018 | India | RCT | Episiotomy only  Spontaneous vaginal delivery only  Infection checked for at 5 days postpartum | 300 women |  |
| 1. Thongtip 2023 | Thailand | Retrospective cross-sectional | All who underwent repair of perineal trauma; infer 1^st^/2^nd^/3^rd^/4^th^ degree tears and episiotomies.  Spontaneous vaginal delivery only  Infection within 72 hours post delivery | 2589 women |  |
| 1. Yilmaz 2024 | Turkey | Prospective observational | Episiotomy only  Spontaneous vaginal delivery  Infection checked for at 1 week, 3 weeks and 1 month post delivery | 400 women |  |

Appendix A: Search terms - An example of the search terms used for MEDLINE.

1. exp Perineum/ and exp Lacerations/

2. exp "Wounds and Injuries"/

3. exp Perineum/

4. 2 and 3

5. Perineum/in [Injuries]

6. ((perineum or perineal) adj3 (trauma* or injur* or wound* or lacerat* or tear* or fissure* or rupture* or damage*)).ab,ti.

7. Anal Canal/in [Injuries]

8. exp Obstetric Labor Complications/

9. 7 and 8

10. (obstetric* and anal and sphincter and (tear* or injur* or trauma or lacerat* or fissure* or wound* or rupture* or damage*)).ab,ti.

11. (OASI or OASIS).ab,ti.

12. exp Episiotomy/

13. episiotom*.ab,ti.

14. exp Vulva/

15. exp "Wounds and Injuries"/

16. 14 and 15

17. ((vulva or vulvar or labial) adj3 (wound* or tear* or rupture* or fissure* or lacerat* or injur* or damage* or trauma*)).ab,ti.

18. ((vagina or vaginal) adj3 (tear* or wound* or rupture* or trauma* or damage* or injur* or lacerat* or fissure)).ab,ti.

19. 16 or 17 or 18

20. 8 and 19

21. 1 or 4 or 5 or 6 or 9 or 10 or 11 or 12 or 13 or 20

22. exp Antibiotic Prophylaxis/

23. ((prophylactic or prophylaxis or preventative or intrapartum or peripartum or antepartum or postnatal) adj3 antibiotic*).ab,ti.

24. 22 or 23

25. 21 and 24

Initial searches in MEDLINE, Embase, Cochrane, CINAHL carried out 30^th^ October 2023 and Web of Science searched 2^nd^ November 2023. The Google Scholar search was carried out on 10th February 2024.The most recent searches in MEDLINE, Embase, Cochrane, CINAHL, Web of Science prior to submission in were carried out 25th June 2024 with results filtered for those up to February 2024.


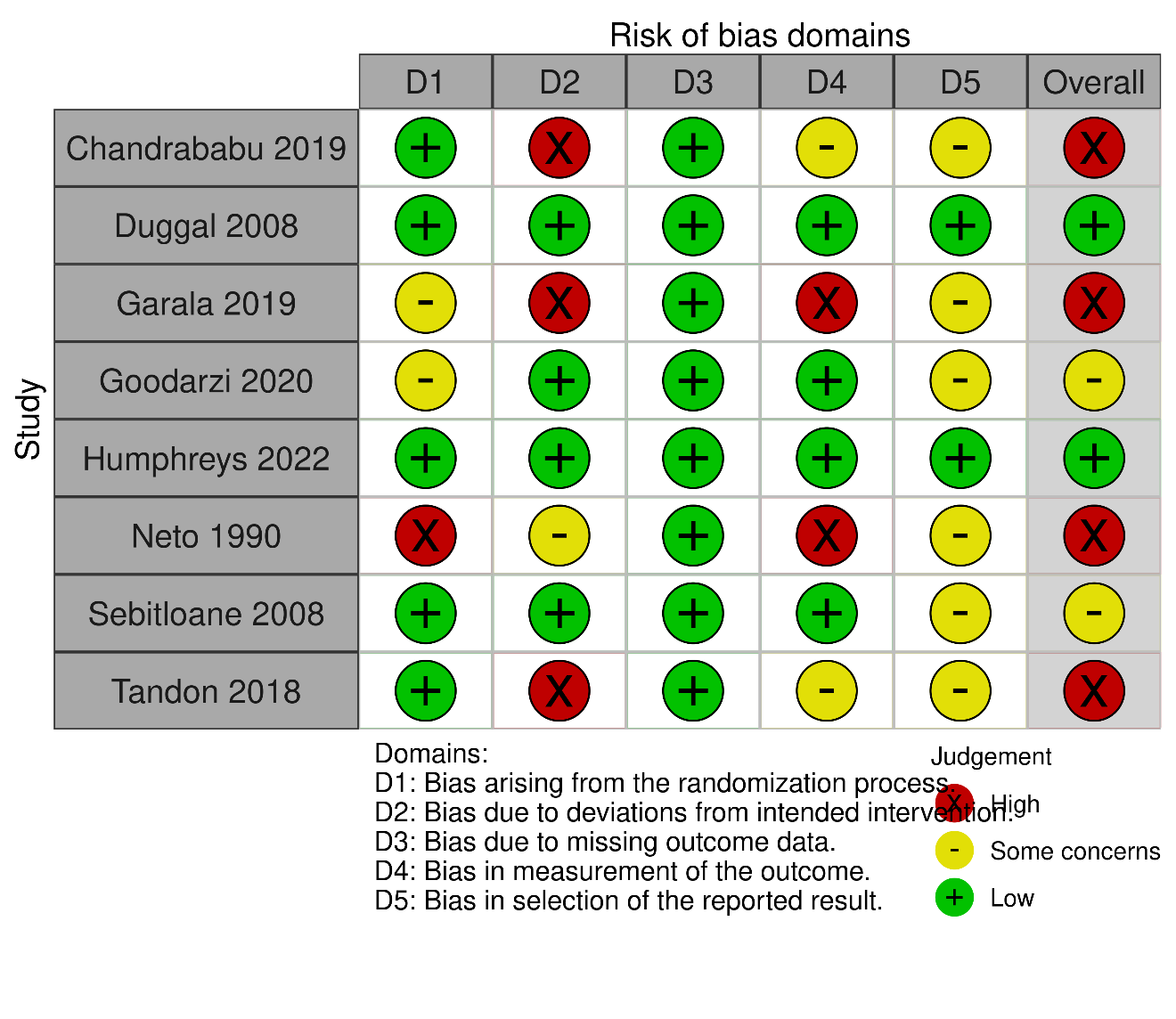


**Domains:**

D1: bias arising from the randomisation process

D2: Bias due to deviations from intended intervention

D3: Bias due to missing outcome data

D4: Bias in measurement of the outcome

D5: Bias in selection of the reported result

**Judgement:**

High (red)

Some concerns (yellow)

Low (green)

Figure A: Risk of bias summary showing the review authors judgement regarding the risk of bias in each for every included RCT at the outcome level. Created using the robivs application. Risk of bias presented at the study level as we found no difference in our risk of bias assessments between the outcomes of wound infection/wound dehiscence within the same study.


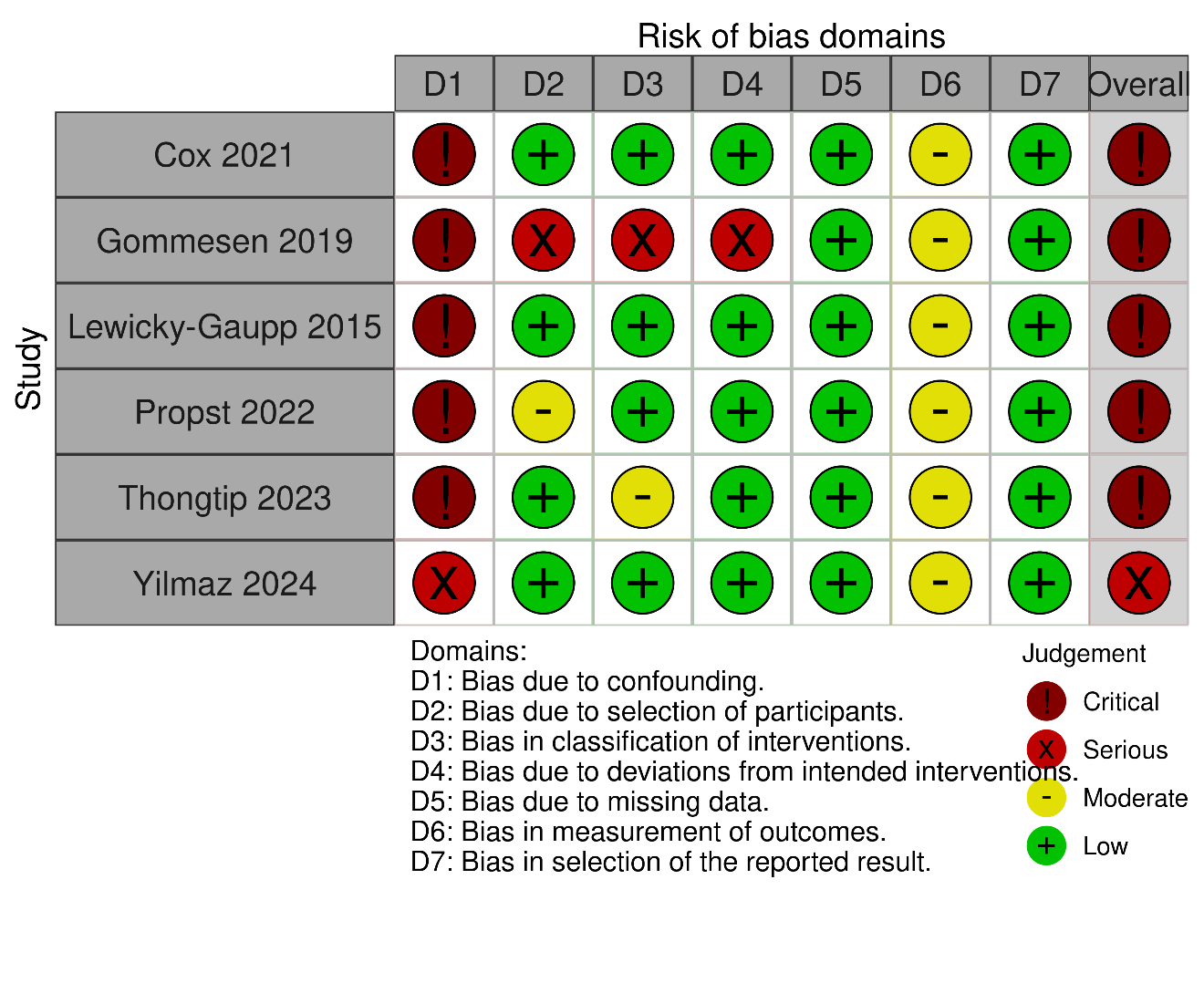


**Domains:**

D1: Bias due to confounding

D2: Bias due to selection of participants

D3: Bias in classification of interventions

D4: Bias due to deviations from intended interventions

D5: Bias due to missing data

D6: Bias in measurement of outcomes

D7: Bias in selection of the reported result

**Judgement:**

Critical: maroon

Serious: red

Moderate: yellow

Low: green

Figure B: Risk of bias summary showing the review authors judgement regarding the risk of bias for included observational studies. Created using the robivs application. We present risk of bias at the study level as we found no difference in our risk of bias assessments between the outcomes of wound infection/wound dehiscence within the same study.

| Table C: GRADE certainty of evidence assessments | | | | | | |
| --- | --- | --- | --- | --- | --- | --- |
| **Prophylactic antibiotics compared to no prophylactic antibiotics for perineal infection** | | | | | | |
| **Population:** all women with perineal trauma  **Intervention**: prophylactic antibiotics  **Comparator:** no prophylactic antibiotics | | | | | | |
|  | **No. of participants (No. of studies)** | **Absolute Risk (95% CI)** | | **Relative Effect (95% CI)** | **Certainty of Evidence (GRADE)** | **Comments** |
|  |  | Prophylactic antibiotics | No prophylactic antibiotics |  |  |  |
| **Outcome: perineal infection** | 3,968 (6) | 66 per 1000  (0.052, 0.083) | 132 per 1000  (0.112, 0.154) | RR 0.51 (0.42, 0.62) | ⊕⊕◯◯  LOW ^1^ | RCT only sensitivity analysis |
| **Outcome: wound dehiscence** | 920 (5) | 58 per 1000 (0.045, 0.074) | 86 per 1000  (0.070, 0.105) | RR 0.77 (0.37, 1.58) | ⊕⊕◯◯  LOW ^2^ | RCT only sensitivity analysis |
| **CI:** confidence interval, **RR:** risk ratio, **GRADE:** GRADE working group | | | | | | |
| **GRADE Working Group grades of evidence**  **High certainty:** Further research is very unlikely to change our confidence in the estimate of effect.  **Moderate certainty:** Further research is likely to have an important impact on our confidence in the estimate of effect and may change the estimate.  **Low certainty:** Further research is very likely to have an important impact on our confidence in the estimate of effect and is likely to change the estimate.  **Very low certainty:** We are very uncertain about the estimate. | | | | | | |
| **Explanations**  ^1^Three studies in this analysis are determined to be at a high risk of bias. Point estimates suggesting effects in the same direction and an I^2^ statistic of 0% demonstrate that significant heterogeneity is likely absent. Differences in the population between studies were present, including a HIV group. The interventions differed across studies, with varying types, routes, doses and durations of antibiotics. Publication bias was not likely present in examination of the funnel plot.  ^2^The proportion of information from studies at high risk of bias is sufficient to affect the interpretation of results. Wide variance of point estimates with I^2^ = 43% indicates moderate heterogeneity. Publication bias was not likely present in examination of the funnel plot. There remain differences in the antibiotic types, routes, dosages and durations. | | | | | | |


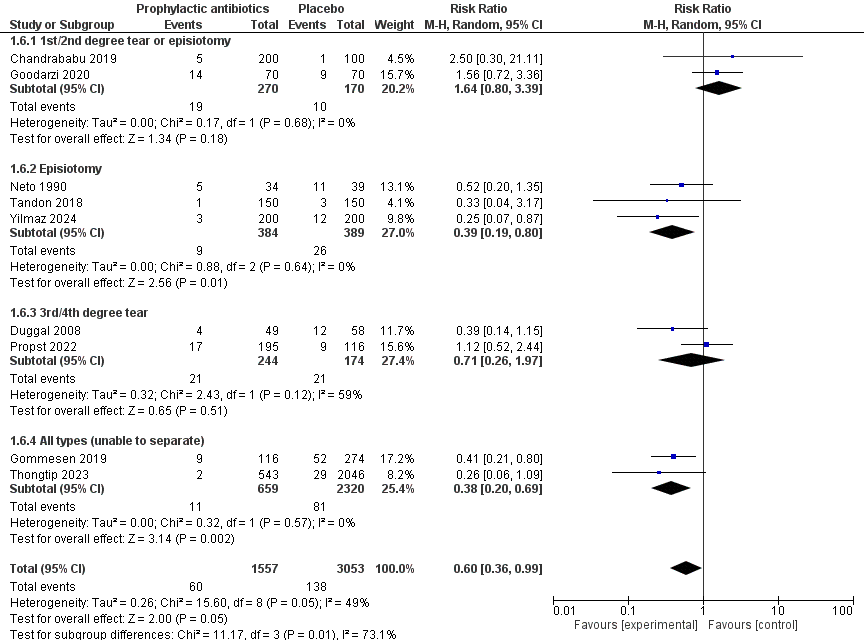


Figure C: Forest plot to show the outcome of wound dehiscence for prophylactic antibiotics compared to control where subgroups are split by type of perineal tear. Created using Revman. Abbreviations M-H=Mantel-Haenszel, CI=confidence interval.


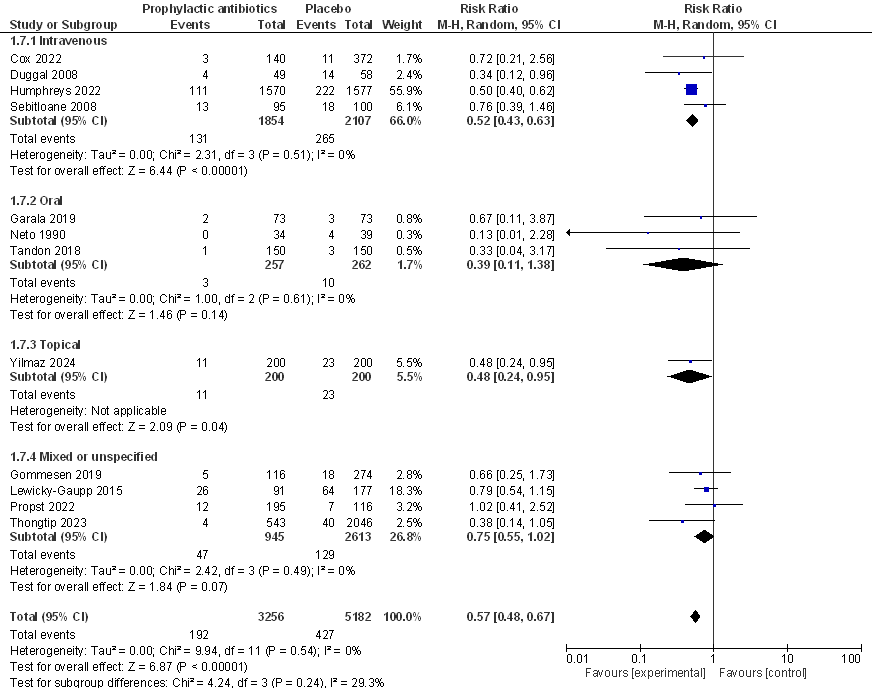


Figure D: Forest plot to show the outcome of wound infection for prophylactic antibiotics compared to control where subgroups are split by route of antibiotic administration. Created using Revman. Abbreviations M-H=Mantel-Haenszel, CI=confidence interval.


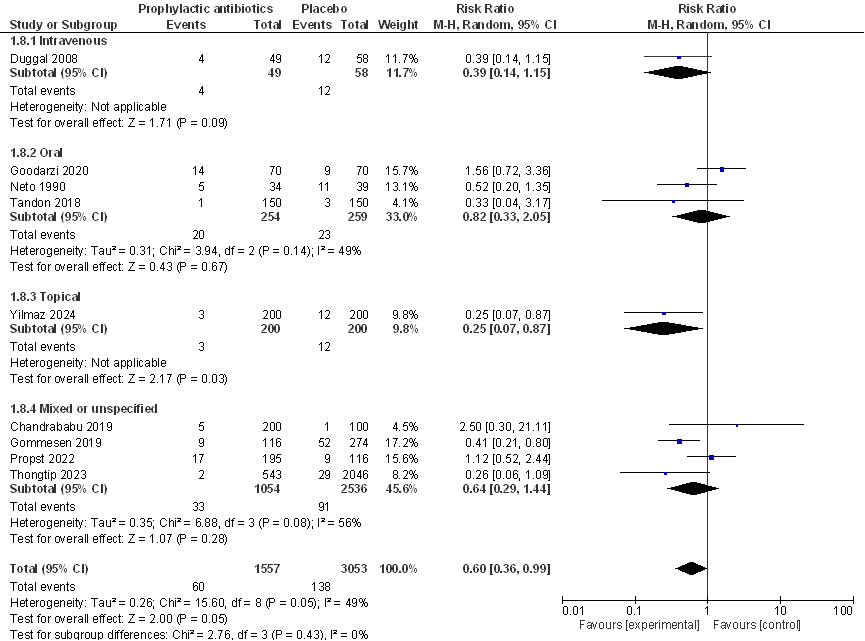


Figure E: Forest plot to show the outcome of wound dehiscence for prophylactic antibiotics compared to control where subgroups are split by route of antibiotic administration. Created using Revman. Abbreviations M-H=Mantel-Haenszel, CI=confidence interval.


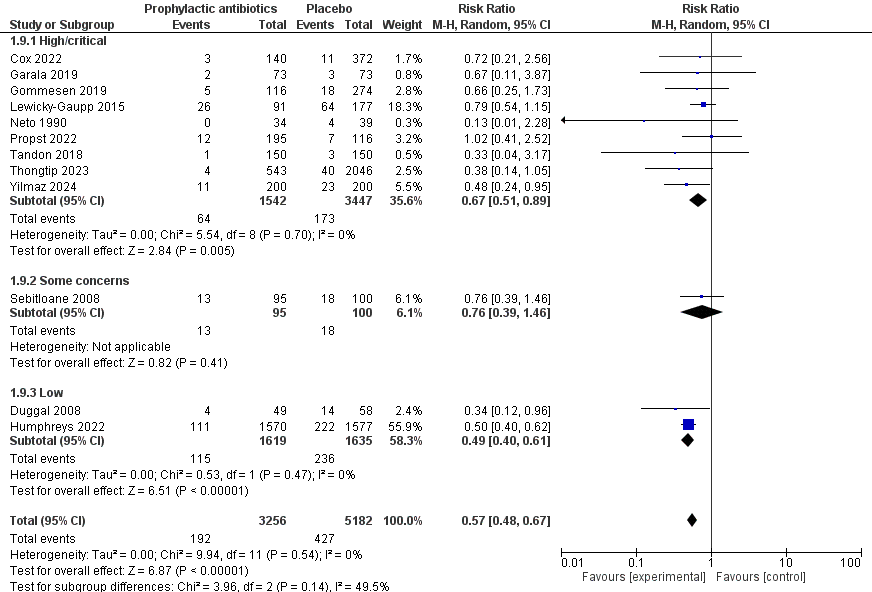


Figure F: Forest plot to show the outcome of perineal wound infection for prophylactic antibiotics compared to control where subgroups are split by risk of bias judgement. Created using Revman. Abbreviations M-H=Mantel-Haenszel, CI=confidence interval.


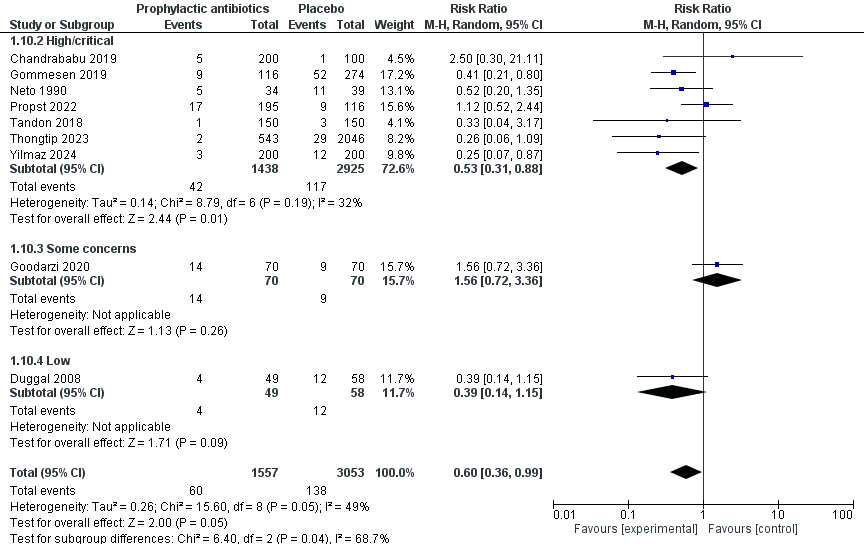


Figure G: Forest plot to show the outcome of perineal wound dehiscence for prophylactic antibiotics compared to control where subgroups are split by risk of bias judgement. Created using Revman. Abbreviations M-H=Mantel-Haenszel, CI=confidence interval.


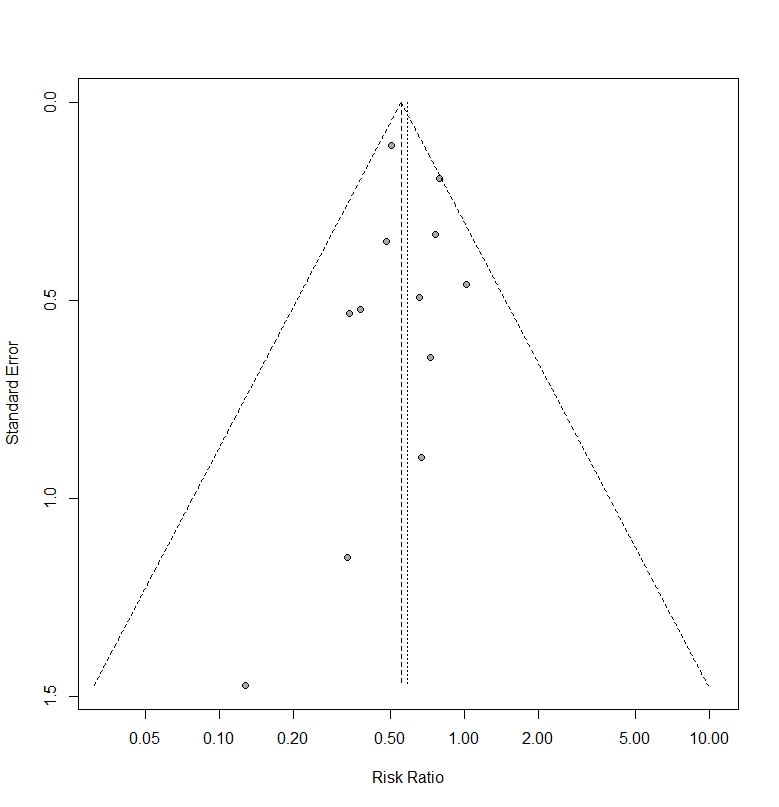


Figure H: Funnel Plot for the outcome of perineal infection.

Eggers' test of the intercept

Intercept= -0.015, 95% CI= -0.89:-0.86, t value= -0.034, p=0.97

Eggers' test does not indicate the presence of funnel plot asymmetry.


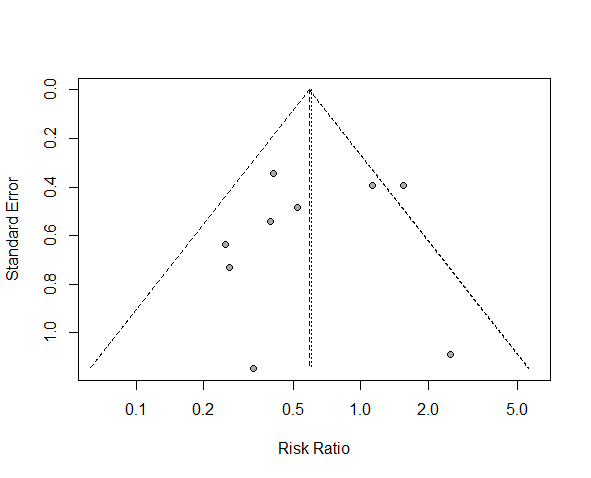


Figure I: Funnel Plot for the outcome of wound dehiscence
